# The Exchange Bias of LaMnO_3_/LaNiO_3_ Superlattices Grown along Different Orientations

**DOI:** 10.1038/s41598-017-11386-8

**Published:** 2017-09-05

**Authors:** Julu Zang, Guowei Zhou, Yuhao Bai, Zhiyong Quan, Xiaohong Xu

**Affiliations:** 1 School of Chemistry and Materials Science of Shanxi Normal University & Key Laboratory of Magnetic Molecules and Magnetic Information Materials of Ministry of Education, Linfen, 041004 China; 2 Research Institute of Materials Science of Shanxi Normal University & Collaborative Innovation Center for Shanxi Advanced Permanent Magnetic Materials and Techonology, Linfen, 041004 China

## Abstract

With the goal of observing and explaining the unexpected exchange bias effect in paramagnetic LaNiO_3_-based superlattices, a wide range of theoretical and experimental research has been published. Within the scope of this work, we have grown high-quality epitaxial LaMnO_3_(*n*)-LaNiO_3_(*n*) (LMO/LNO) superlattices (SLs) along (001)-, (110)-, and (111)-oriented SrTiO_3_ substrates. The exchange bias effect is observed in all cases, regardless of growth orientation of the LMO/LNO SLs. As a result of a combination of a number of synchrotron based x-ray spectroscopy measurements, this effect is attributed to the interfacial charge transfer from Mn to Ni ions that induces localized magnetic moments to pin the ferromagnetic LMO layer. The interaction per area between interfacial Mn and Ni ions is nearly consistent and has no effect on charge transfer for different orientations. The discrepant charge transfer and orbital occupancy can be responsible for the different magnetic properties in LMO/LNO superlattices. Our experimental results present a promising advancement in understanding the origin of magnetic properties along different directions in these materials.

## Introduction

Transition-metal oxides have long been a major focus of condensed-matter research due to their strong correlation between charge, spin, lattice and orbital degrees of freedom^1^. These artificial superlattices provide a wealth of properties not present in traditional materials, such as interfacial superconductivity observed between insulators LaAlO_3_ and SrTiO_3_ and ferromagnetism at the interface of antiferromagnetic CaMnO_3_ and paramagnetic CaRuO_3_^2, 3^. Recently, paramagnetic metal LaNiO_3_-based heterostructures have inspired a lot of research, largely due to their antiferromagnetism and the possibility of stable high-temperature superconductivity that has basis in theoretical prediction^4, 5^. Taking Gibert *et al*. as an example, the exchange bias (EB) effect was observed in (111)-oriented superlattices that consist of ferromagnetic LaMnO_3_ (LMO) and paramagnetic LaNiO_3_ (LNO), while it was not observed in SLs grown along the (001) direction^6^. This compelling phenomenon has spurred a wide range of theoretical and experimental work seeking to explain diverse exchange bias in different orientations of the SLs^7–12^. Using tight-binding calculations, Dong *et al*. have found that magnetism in (111) is higher than in (001) direction due to quantum confinement, independent of the charge transfer^7^. However, using density functional theory calculations from first principles, Lee *et al*. have found the magnetic moments are induced by charge transfer between interfacial Ni and Mn ions and are similar for the (001) and (111)-oriented SLs^8^. Indeed, the large charge transfer has been observed by Hoffman *et al*. in (001)-oriented superlattices^9^. Furthermore, Zhou *et al*. have recently indicated the absence of exchange bias in the relatively thick LMO/LNO superlattice along (001) orientation is due to charge transfer being suppressed by orbital reconstruction^13^. In addition, it was found that the exchange bias can be observed in thin LMO/LNO superlattice along the (001) direction^14^. However, these remarkable findings indicate that information required to analyze the relationship between exchange bias effect and various orientations of LMO/LNO superlattices is still lacking.

Within the scope of this research, we have grown LMO/LNO superlattices along various orientations to investigate the relationship between interfacial structure, magnetic behavior, charge transfer, and orbital occupancy using X-ray absorption spectroscopy (XAS) and X-ray linear dichroism (XLD) measurements. Regardless of LMO/LNO SL growth orientation, the unexpected exchange bias is still observed due to the charge transfer from Mn to Ni ions causing localized magnetic moments that pin the ferromagnetic LMO layer. Diverse charge transfer and orbital occupancy of e_g_(*x* ^2^ − *y* ^2^) for (001) SL and e_g_(*3z* ^2^ − *r* ^2^) for (110) SL are found to be responsible for the variable magnetic properties.

## Results and Discussion

**Figure 1:**
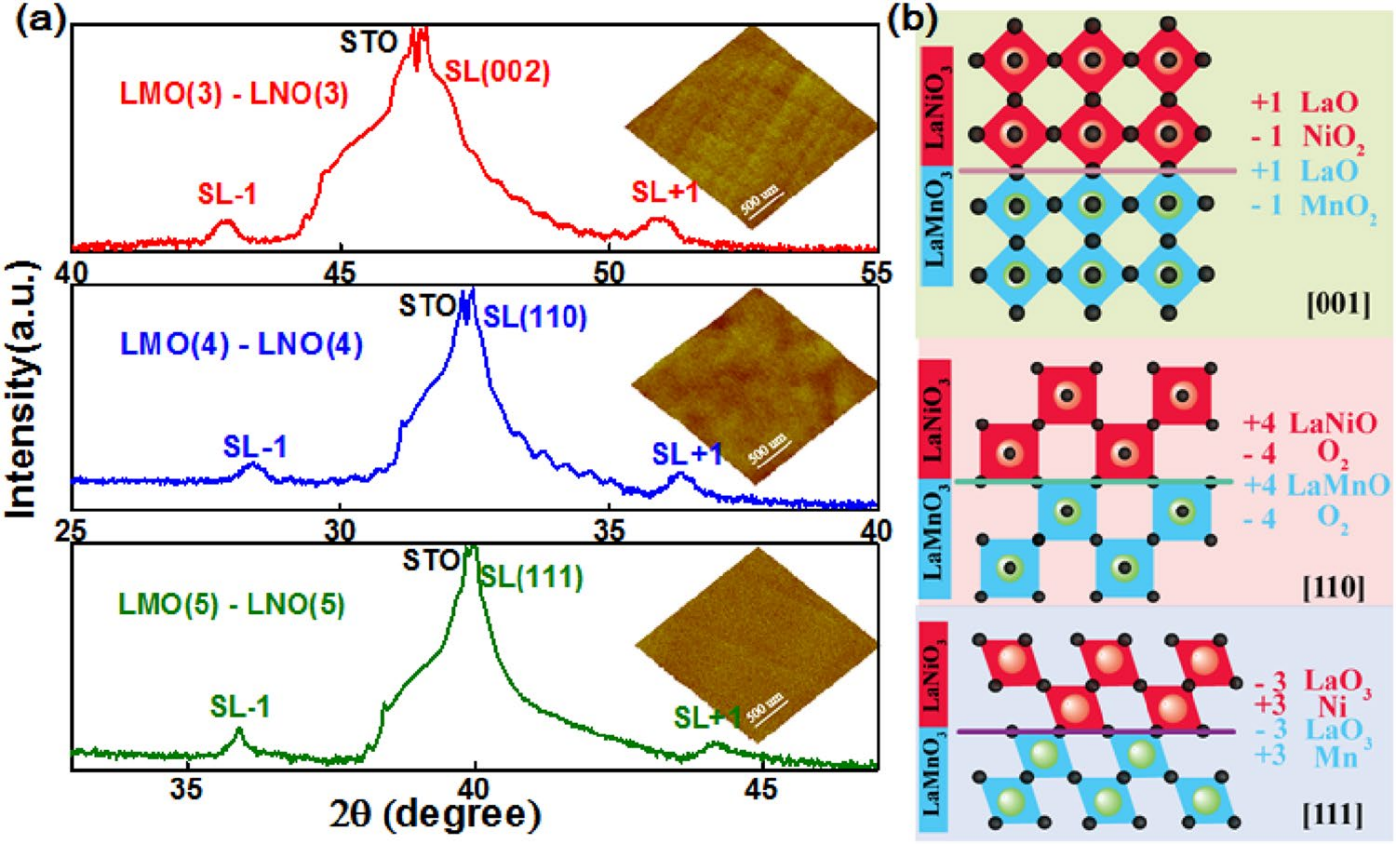
(**a**) XRD patterns for (3–3)_10_, (4–4)_10_, and (5–5)_10_ LMO/LNO SLs grown along different directions of (001), (110), (111) STO substrates, respectively. Note the satellite peaks around the different directions of the Bragg reflection. Surface topographies of LMO/LNO SLs in different directions are shown in the inset. (**b**) Schematics of structure and polarity along [001], [110], and [111] directions for LMO/LNO SLs, respectively.

To gain insight to the structure along different directions of LMO/LNO SLs, we recorded X-ray diffraction patterns using Cu Kα radiation. This lead to several key observations presented here. As shown in Fig. 1(a), the out-of-plane crystallographic directions of SLs are determined to be (001), (110) and (111) with respect to the reflections around symmetric peaks of (002), (110) and (111), respectively. The double-peak structures of different STO substrates are caused by Kα_1/2_ splitting of the incident X-ray beam during the measurement^15^. Furthermore, the main peak of the superlattice is obscured by the intensity peak of the STO substrate due to the relatively low total thickness of different SLs (23 nm). However, the satellite peaks (SL + 1 and SL − 1) are observed in all directions and suggest smooth interfaces between the LMO and LNO layer^16^. In the inset of Fig. 1(a), the surface roughness is shown to be 0.094 nm for (001) SL, 0.156 nm for (110) SL, and 0.142 nm for (111) SL by atomic force microscopy (AFM) measurements, respectively. Dramatic differences of LMO/LNO interfaces in the ionic arrangement of (001), (110) and (111) planes are shown in Fig. 1(b). In the [001] direction, the stacking of LaO^1+^ and NiO_2_^1−^ (or MnO_2_^1−^) in LNO (or LMO) layer exhibit in-plane alternating charged planes of 1+ and 1−, making [001] direction weakly polar. However, in the other directions ([110] or [111]), the stacking of LaNiO^4+^ (LaO_3_^3−^) and O_2_^4−^ (Ni^3+^) in LNO layer makes [110] and [111] directions highly polar. Therefore, growth of LMO/LNO SLs along (110) and (111) directions likely requires more severe compensation mechanisms beyond the (001) direction, such as additional chemical, structural, or electronic reconstruction^17^. Furthermore, in the case of SLs grown along the [001] orientation, each interfacial B cation (Mn and Ni) has one of the other B’ species and five of the same B-cation neighbors. The interfacial B cation has two of the other B’ cations as neighbors along [110] direction, and three of them along [111] direction. Therefore, naively, the interaction between interfacial Mn and Ni ions in (110) and (111) planes could be expected to be enhanced in comparison to the (001) stacking^6, 7^. However, this scenario is likely too simplistic. The interfacial competition with various orientations of SLs is compared in the following section.

**Figure 2:**
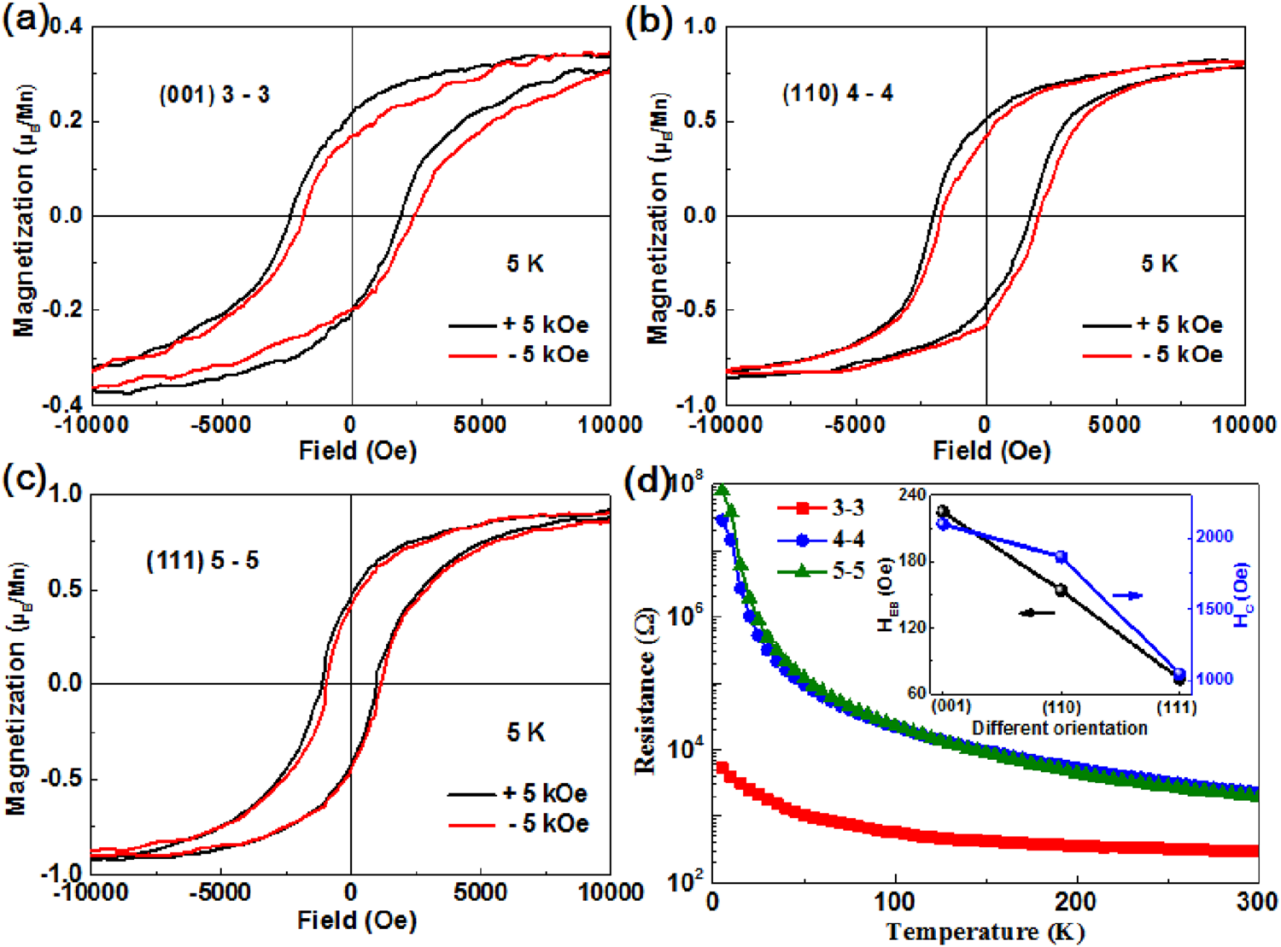
Magnetic hysteresis loops of 3–3 (**a**), 4–4 (**b**), and 5–5 (**c**) LMO/LNO SLs measured along in-plane direction at 5 K after ±5 kOe field cooling from room temperature, respectively. (**d**) Temperature dependence of the resistance for different orientation of LMO/LNO SLs. The inset shows the variation of *H*_*EB*_ and *H*_*C*_ dependence for various directions.

Gibert *et al*. have reported an unexpected exchange bias effect in the (111)-oriented (7–7) superlattices and declared the absence of EB in (001)-oriented (7–7) SLs of LMO/LNO systems^6^. However, Zhou *et al*. have recently observed exchange bias in the relatively thin layer in (001) stacking of LMO/LNO SLs and found that thicker SLs exhibit lower charge transfer rates compared to thinner SLs^13^. Inspired by these findings, we chose the thin SLs as (3–3)_10_ in (001) plane, (4–4)_10_ in (110) plane, and (5–5)_10_ in (111) plane for comparison. In these cases, we controlled the total thickness of three different orientations of LMO/LNO SLs (around 23 nm). Magnetic properties were measured with the field applied in-plane with respect to the SLs. Fig. 2(a) shows the hysteresis loops of (001)-oriented superlattice at 5 K after ±5 kOe field-cooling (FC) processes started at room temperature. A shift along the magnetic field axis and enhanced coercivity were observed simultaneously in the FC loop. These features are caused by the exchange bias effect^18^. The EB effect can be quantitatively described by the formulas *H*_*EB*_ = |(*H*_*1*_ + *H*_*2*_)/2| and *H*_*C*_ = |(*H*_*1*_ − *H*_*2*_)/2|, where *H*_*1*_ and *H*_*2*_ are the negative coercive field and the positive coercive field at which the magnetization equals zero, respectively^19^. As shown in this figure, the highlighted negative coercive field is −2322 Oe and the positive coercive field is 1872 Oe. Therefore, the exchange bias field of 225 Oe and coercive field of 2097 Oe are derived from the +5 kOe field-cooling loop of the (001)-oriented LMO/LNO superlattice. In contrast, on cooling in a −5 kOe filed, a shift of the center of the magnetic loop along the magnetic field axis was observed towards positive fields. In the case of (110) and (111) directions, the EB effect caused by the field cooling process is observed to be 154 Oe and 74 Oe, as shown in Fig. 2(b) and (c). This behavior indicates that the EB seems to be an intrinsic property for LMO/LNO superlattices and is independent of the crystallographic direction, consistent with previous theoretical predictions^8^. The variation in *H*_*EB*_ and *H*_*C*_ relationship for different stacking directions of LMO/LNO SLs is shown in the inset of Fig. 2(d). Direct current transport measurements for different orientations of the SLs are shown in Fig. 2(d), where the insulating behavior is still observed. This feature is attributed to the insulating character of thinner LNO-based superlattices, induced by the reduced dimensionality^20^. The resistance of the (001)-oriented SLs is clearly diminished by about one order of magnitude at room temperature in comparison with that of (110) and (111) directions.

**Figure 3:**
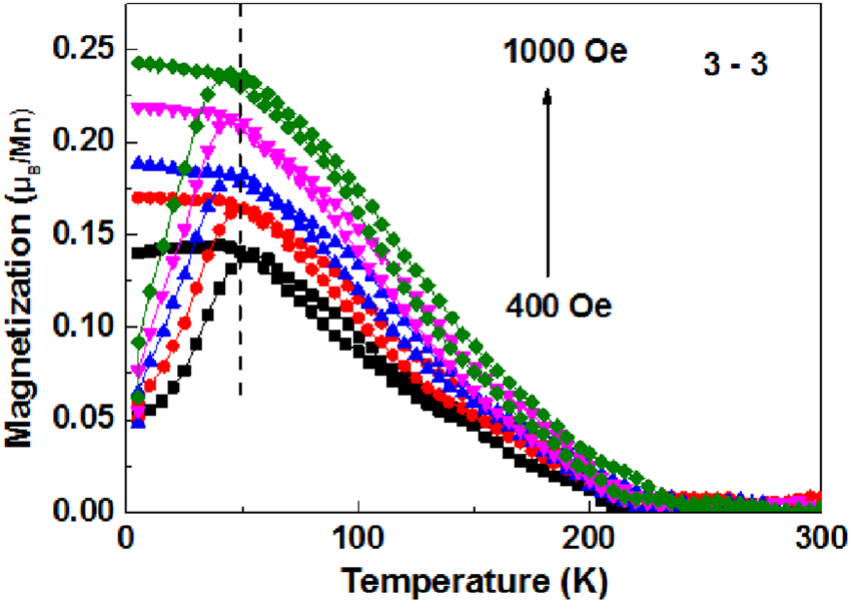
*M*-*T* curves of the 3–3 superlattice measured under different magnetic fields after ZFC and FC process.

In order to exclude the spin glass state in our experiment, we have measured magnetization versus temperature (*M-T*) curves under various fields of 400, 500, 600, 800, and 1000 Oe after FC and ZFC processes in Fig. 3. The peak in the ZFC curves (*T*_*P*_) and a bifurcation between the ZFC and FC curves below the irreversibility temperature (*T*_*irr*_) are previously observed in a spin glass based exchange bias system^18^. However, for the spin glass system, both temperatures are greatly reduced upon increasing measurement field, suggesting that the frozen state is clearly suppressed by a strong field^15^. In our experiment, the two characteristic temperatures are nearly constant when the measurement field is increased, thereby excluding the existence of spin glass behavior.

**Figure 4:**
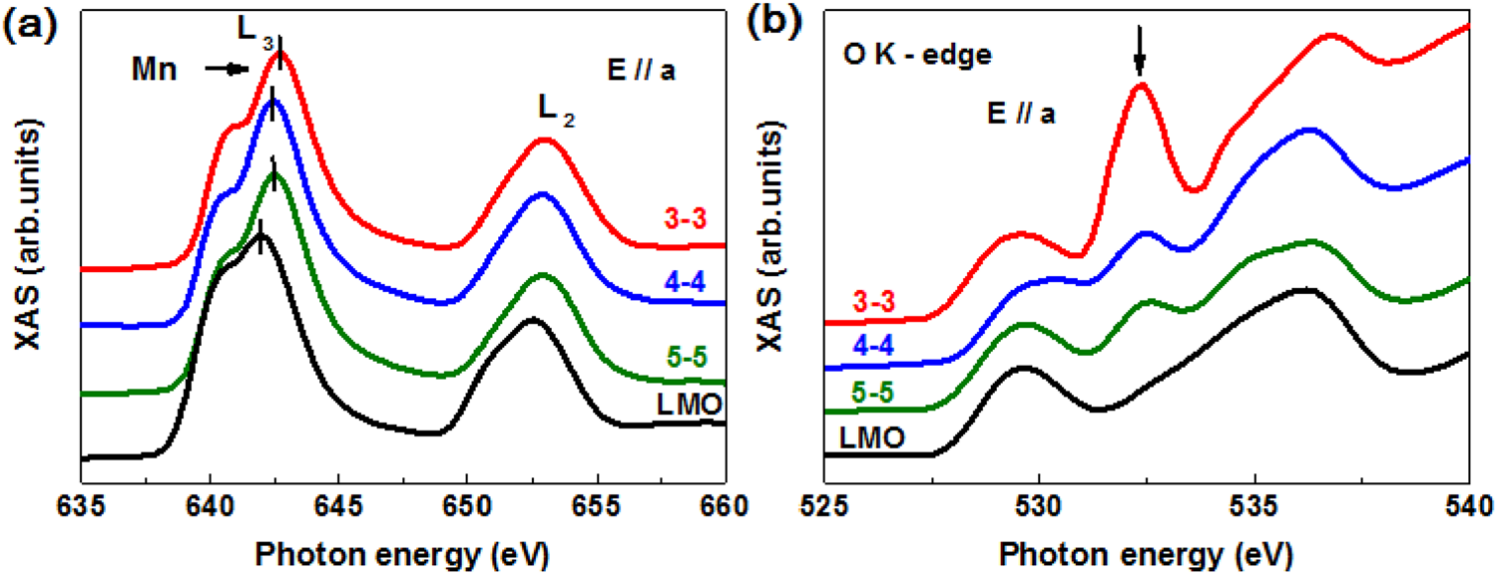
Normalized XAS spectra at the (**a**) Mn *L*-edge and (**b**) O *K*-edge from different samples recorded in TEY mode at room temperature. Arrows mark the variation around the Mn *L*-edge and O *K*-edge in different LMO/LNO SLs. The spectra are vertically offset to allow better visualization.

In order to explore the origin of this disparate magnetic behavior and transport properties in SLs, we performed a variety of X-ray absorption spectroscopy measurements. Fig. 4(a) shows Mn *L*-edge XAS for various directions of LMO/LNO SLs with the single LMO film for reference. The XAS at the Mn *L*-edge can provide important information about the unoccupied Mn 3*d* state and related Mn valence due to the sensitivity of Mn 2*p*_3/2, 1/2_ → 3*d* dipole transitions^21^. Furthermore, because of the separation originating from the spin-orbit splitting of the Mn 2*p* core hole, the spectrum contains broad multiplets: the Mn *L*_3_ peak (low energy) and the *L*_2_ peak (high energy). The most striking finding here is the shift of *L*_3_ peak toward higher energy in comparison with LMO single film. From the published spectra of Mn XAS on La_*1−x*_Sr_*x*_MnO_3_, it is known that the shift of the XAS spectrum towards higher energies is mainly due to Mn^4+^ ^22^. Comparison of the published spectra with our data reveals that the (3–3) SL clearly shows more Mn^4+^ valence than other two directions of SLs, while the Mn spectral features for (4–4) and (5–5) indicate a valence state between those of LMO and the (3–3) SL. Moreover, the presence of more Mn^4+^ valence in (3–3) SL is further supported by the O *K*-edge, as displayed in Fig. 4(b). The XAS at the O *K*-edge can supply useful additional information on the Mn 3*d* occupancy due to the hybridization between the interfacial Mn and Ni ions through the O 2*p* states^23^. The O *K*-edge is mainly influenced by the unoccupied O 2*p* states via O 1 *s* → 2*p* transition^24^. Because the peak of 536 eV influenced by the La 5*d* orbit, the pre-edge from 528 to 534 eV and principally the peak of 533 eV are marked in Fig. 4(b) to show the O 2*p* orbital is hybridized with mixed electrons of Mn or Ni ions. The peak at 533 eV is distinctly visible in all SLs, and its intensity is larger in the (001)-oriented SL, indicating the higher degree of hybridization between Mn and Ni ions. As the Fermi level of LMO is higher than that of LNO layer, the presence of electron transfer from Mn to Ni sites is in good agreement with the results of Mn *L*-edge and O *K*-edge spectra^13^. Therefore, it is plausible to consider the charge transfer occurring from interfacial Mn to Ni ions in SLs.

**Figure 5:**
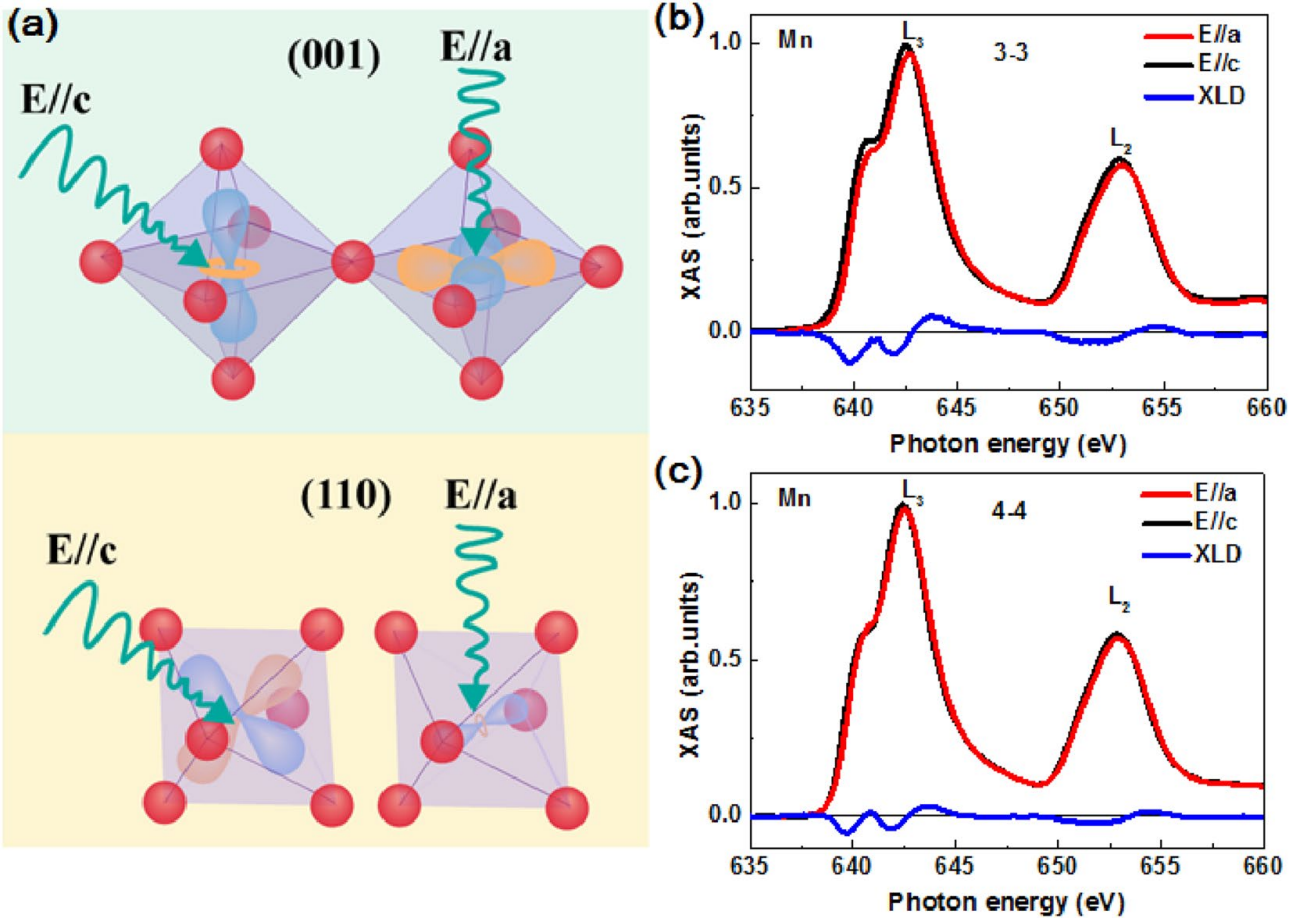
(**a**) Schematic representations of the polarized X-ray linear dichroism measurements for both (001) and (110) directions LMO/LNO superlattices with in-plane (E//a) and out-of-plane polarization (E//c). Normalized XAS and XLD for different LMO/LNO SLs are shown in (**b**) 3–3 and (**c**) 4–4.

In transition metal-oxides heterostructures, the variation of orbital degree of freedom is known to generate a multitude of electronic phases with radically different macroscopic properties, as reported for LaNiO_3_/LaAlO_3_ system^25^. However, for various LMO/LNO orientations, there is still a scarcity of adequate information about the orbital occupancy at the interface. Based on the excitation of core electrons into the valence *d* orbitals employing linearly polarized photons, XLD is the only method that can determine the spatial average of orbital occupation^26^. Fig. 5(a) shows the schematic diagrams of measurements in (001) and (110)-oriented LMO/LNO SLs at the BL08U1A beamline in total electron yield mode. Photon polarization during the measurement is parallel to the sample plane (E//a) and is almost perpendicular to the sample plane (E//c) for various SLs. XLD is calculated as the difference of intensities between the XAS in-plane and out-of-plane components, in order to determine the occupancy of Mn 3*d* orbits^27^. In (001)-oriented SLs, the in-plane and out-of-plane components are proportional to hole occupancies for the e_g_(*x* ^2^ − *y* ^2^) and e_g_(*3z* ^2^ − *r* ^2^) orbits. Conversely, the in-plane and out-of-plane components are proportional to the hole occupancies for the e_g_(*3z* ^2^ − *r* ^2^) and e_g_(*x* ^2^ − *y* ^2^) orbits in (110)-oriented SL. The positive/negative area under XLD (*I*_//_ − *I*_*⊥*_) is due to the preferential occupancy of e_g_(*3z* ^2^ − *r* ^2^)/e_g_(*x* ^2^ − *y* ^2^) in SL of the (001) orientation and is inverse with respect to the preferential occupancy of the e_g_(*x* ^2^ − *y* ^2^)/e_g_(*3z* ^2^ − *r* ^2^) in (110) orientation. However, in the (111) direction SL, orbital occupancy should be probed by X-rays impinging at a grazing incidence of 54.7° and 35.3° onto the measured sample, which is omitted in this paper. Fig. 5(b) and (c) show the XLD spectra as well as the in-plane and out-of-plane XAS spectra for different directions of SLs, respectively. The XLD spectral area of (001)-oriented SLs is negative, indicating that the tensile strain of STO substrate results in a preferential occupancy of the relatively low energy e_g_(*x* ^2^ − *y* ^2^) orbit. Furthermore, the XLD spectral area of (110) direction SLs is also negative, implying the preferential occupancy of the lower energy of e_g_(*3z* ^2^ − *r* ^2^) orbit according to the schematic diagrams. This finding is in agreement with the analysis published by Fontcuberta *et al*.^28^.

The correlation between structure, magnetic behavior, transport properties and charge transfer in different orientations of LMO/LNO superlattices was also investigated. In agreement with previous published results, when the LNO layer becomes thicker and reestablishes its bulk-like metallicity, the highly polar discrepancy can be easily avoided by metallic screening^29^. In this research, the insulating states of various SLs are confirmed by transport measurements and the highly polar (110) and (111) orientations are compensated by the relatively rough interface structure in AFM measurements. In agreement with previous results, high quality nickelates still present a challenge due to their highly polar atomic layers along the [110] and [111] directions^17^. Considering the interaction of interfacial Mn and Ni cations, every Mn ion along [111] or [110] direction has triple (or double) coupling interaction strength with Ni species compared to the [001] direction. However, the estimated in-plane Ni-Ni planar distance is 3.84 Å, 5.23 Å, and 6.65 Å for (001), (110), and (111) directions of the LNO layer, respectively. The total interaction per area around the interfacial B-B’ cations can be tracked with the relation *N*/*S* = *t*/*d* ^2^, *t* is the number of different B’ cations, and *d* is the in-plane B-B planar distance in various directions^30^. Therefore, it can be concluded that in different SL directions the interfacial interaction per area between Mn-Ni cations is approximately equal, having no contribution to the charge transfer. Previous research found that the charge transfer in interfacial Mn and Ni ions can result in local magnetic moment pinning the ferromagnetic LMO layer and causing exchange bias in LMO/LNO SLs^31^. Regarding the degrees of freedom of the charged particles, electric transport provides direct means of controlling the energy of this element in the ground state^32^. The charge transfer in the (001)-oriented SL is larger than in the other ((110) and (111)) directions, supported by the polar discrepancy and transport measurements in different orientations. Thus, the stronger exchange bias in the (001) direction is attributed to the larger charge transfer between interfacial Mn and Ni ions. In order to further elucidate the orbital occupancy effect on magnetic properties, in-plane e_g_(*x* ^2^ − *y* ^2^) for (001) SL and e_g_(*3z* ^2^ − *r* ^2^) for (110) SL occupancies are preferential, which is consistent with the effect of tensile strain^33^. This phenomenon is likely to be responsible for the difference in magnetism of (001) and (110) oriented SLs. However, the specific mechanism is presently not fully understood and requires further investigation.

## Conclusions

The relationships between structure, magnetic behavior, charge transfer and orbital occupancy have been investigated for different orientations in LMO/LNO superlattices using XAS and XLD measurements. For different orientations of SLs, the interaction per area between interfacial Mn and Ni ions is found to be very similar for constant areas. However, unexpected exchange bias was observed in different orientations of LMO/LNO superlattices. This effect can be explained by charge transfer from Mn to Ni ions that induce localized magnetic moments that pin the ferromagnetic LMO layer. The discrepant orbital occupancy of e_g_(*x* ^2^ − *y* ^2^) for (001) SL and e_g_(*3z* ^2^ − *r* ^2^) for (110) SL, as well as the diverse charge transfer, play a crucial role in various magnetic phenomena in LMO/LNO superlattices. Our findings present a promising advancement in understanding of the origin of magnetic properties along various directions in superlattices.

### Experimental details

High quality epitaxial [LaMnO_3_(*n*)-LaNiO_3_(*n*)]_10_ superlattices were grown by a pulsed laser deposition system (PLD) and probed with *in-situ* reflection high energy electron diffraction (RHEED). The *n* indicates the number of unit cells (u.c.), hereafter referred to as (*n-n*)_10_ SLs. The SrTiO_3_ (STO) single crystals with (001), (110) and (111) orientations were selected as substrates. In order to achieve approximately the same total thickness, *n* was chosen to be 3 monolayers in (001), 4 monolayers in (110), and 5 monolayers in (111), motivated by the estimated out-of-plane Ni-Ni planar distance as 3.84 Å, 2.72 Å, and 2.22 Å, respectively. Before the PLD deposition, the substrates were etched with a NH_4_F buffered HF solution and subsequently annealed in an oxygen atmosphere in order to obtain atomically flat substrate surfaces^34^. The growth was directed downward at 725 °C substrate temperature and 100 mTorr oxygen environment, using a KrF excimer laser (λ = 248 nm) with 2 Hz repetition rate and 330 mJ energy. In order to avoid further oxygen vacancies, the SLs were annealed *in-situ* in 300 Torr oxygen pressure at growth temperature for 1 hour after deposition. Out-of-plane crystal structures in different orientations of the SLs were examined using X-ray diffraction (XRD) and their magnetic properties were measured by a superconducting quantum interference device (SQUID). In order to exclude the influence from the remnant magnetization of the superconducting magnet, we have measured the standard sample (Pd) in SQUID and obtained the remnant magnetization as -2.2 Oe. When measuring the magnetic hysteresis loops of the LMO/LNO superlattices, the magnetic field was set to 2.2 Oe instead of 0 Oe to correct the remnant magnetization of the superconducting magnet. In-plane resistance was measured as a function of temperature in four-point van der Pauw geometry by a physical properties measurement system (PPMS). X-ray absorption spectroscopy and X-ray linear dichroism measurements were performed at room temperature in total electron yield (TEY) mode by soft X-ray regime at the Mn *L*-edge and O *K*-edge absorption edge at the Shanghai Synchrotron Radiation Facility (SSRF) and National Synchrotron Radiation Laboratory (NSRL), China. The XLD signals were determined by the difference between the XAS in-plane component E//a (with the X-rays impinging at 90 degrees with respect to the sample), and out-of-plane E//c (with X-rays impinging at 30 degrees grazing incidence with respect to the sample).
